# Youth Engagement in School Mental Health Teaming: Structure, Processes, and Outcomes of a Youth Leadership Academy to Promote Emotional Well-Being in Schools

**DOI:** 10.3390/bs15111563

**Published:** 2025-11-17

**Authors:** Tiffany S. Beason, Zahra Ladhani, Perrin Robinson, Kathryn M. Trainor, Jenna E. Russo, Jessica Bernstein, Jill H. Bohnenkamp

**Affiliations:** 1 Department of Psychiatry, Division of Child and Adolescent Psychiatry, School of Medicine, University of Maryland, Baltimore, 620 W Lexington St., Baltimore, MD 21201, USAjbohnenk@som.umaryland.edu (J.H.B.); 2 College of Education, University of Florida, Gainesville, FL 32611, USA; ktrainor@coe.ufl.edu; 3 Department of Psychology, Mississippi State University, 75 B.S. Hood Drive, Starkville, MS 39762, USA; 4 Collaborative for Academic, Social, and Emotional Learning, 815 W. Van Buren St., Suite 210, Chicago, IL 60607, USA

**Keywords:** youth engagement, youth leadership academy, school mental health, positive youth development, school mental health teaming, K-12 schools, youth leadership, universal mental health promotion in schools, multi-tiered system of supports (MTSS)

## Abstract

Background: It is essential that leaders in education and behavioral health partner with youth to build Comprehensive School Mental Health (CSMH) systems. One mechanism to elevate youth perspectives in CSMH system building is by engaging youth as members of CSMH teams. Method: The current study describes the structure, process and impact of a school-based *Youth Leadership Academy* (YLA) that integrated youth leaders into CSMH teams with state, district, and school leaders. The YLA offered student leaders opportunities to (1) receive training and mentorship to enhance their capacities to serve as leaders on CSMH teams, (2) provide input on CSMH priorities and (3) set MTSS goals to advance emotional well-being in schools. This study summarizes youth-driven multi-tiered systems of support (MTSS) goals and action plans by youth participants and the impact of youth participation in the YLA on indicators of positive youth development. Results: Youth most often contributed to planning and/or implementation of Tier 1-Universal Mental Health supports related to mental health literacy and school climate. Pre-post surveys revealed YLA participation was associated with statistically significant increases in youth reports of core social and emotional learning skills, positive identity, and contribution. Conclusions: Schools can replicate the YLA structure to enhance MTSS and foster youth leader skill development.

## 1. Introduction

Comprehensive school mental health (CSMH) systems are an established path to both responding to rising rates of youth mental health concerns and promoting well-being for all students (Hoover et al., 2019). Schools typically implement CSMH systems using a multi-tiered system of support (MTSS). MTSS tiers include Tier 1—Universal Mental Health Promotion/Prevention supports (e.g., social and emotional skills cultivation, positive school climate, positive disciplinary practices, mental health literacy), Tier 2—Early Intervention (e.g., small group counseling), and Tier 3—Treatment (i.e., individual and family therapy) (Hoover et al., 2019; Weist et al., 2018). Tiered school-based mental health supports are associated with improvements in student social–emotional skills and mental health (Colomeischi et al., 2022) along with enhanced academic engagement and school climate (Hoover & Bostic, 2021; Golberstein et al., 2023).

It is best practice that youth, families, and state and local leaders in education, behavioral health, and all systems affecting children’s mental health work together on coordinated cross-systems teams to build CSMH systems (Miles et al., 2010). Once established, cross-system CSMH teams benefit from receiving guidance from national school mental health training and technical assistance (TTA) centers (e.g., the National Center for School Mental Health, NCSMH) via Learning Collaboratives (Bohnenkamp et al., 2024). Learning collaboratives (LC) often function as Communities of Practice, where cross-system CSMH teams benefit from opportunities to collaborate across systems and learn from the successes and lessons learned from other states. Teams can use these resources to establish a unified vision and mission statement as well as goals and corresponding action plans to support continuous quality improvement of CSMH systems (Bohnenkamp et al., 2024). This emphasis on coordinated efforts aligns with the Whole School, Whole Community, Whole Child (WSCC) model, which offers a framework for integrating education, health, and community systems to support the whole child (Centers for Disease Control and Prevention, 2015; Lever et al., 2024).

In a recent evaluation of multi-level national learning collaboratives, youth partnership emerged as particularly influential to improvements in CSMH system quality among LC participants (Bohnenkamp et al., 2024). However, youth engagement in CSMH system building has historically lacked both depth and integration. In early initiatives, young people were sometimes invited to participate only after programmatic decisions had already been made. These symbolic forms of involvement did little to shift the power imbalance or elevate youth perspectives. Hart’s (1992) ladder of participation remains a widely cited framework for understanding this issue, outlining a continuum from manipulation and tokenism to youth-initiated, shared decision-making. While awareness of the importance of meaningful youth engagement continues to grow, many CSMH efforts operate at the lower rungs of this ladder.

There are emerging models that reflect deeper youth engagement. One example is *Bring Change 2 Mind* (BC2M), a national high school club model that promotes mental health awareness through peer-led education. Evaluations of BC2M have shown it can improve students’ attitudes toward mental health and reduce stigma (Fein et al., 2023). Yet, BC2M clubs typically operate as stand-alone programs rather than being embedded within school-wide mental health systems. This illustrates a broader pattern in which youth involvement, while expanding, remains fragmented and peripheral to the core structure of CSMH systems.

Research consistently demonstrates the benefit of meaningful youth engagement. When young people are meaningfully involved, mental health programs become more culturally responsive, relevant, and effective. Youth provide unique insight into peer experiences and needs, which helps ensure that interventions resonate with the communities they aim to serve (McCabe et al., 2023). Engagement also contributes to positive school climates. Fein et al. (2023) found that BC2M student leaders helped foster more inclusive and stigma-free environments. In addition, youth participation supports the development of leadership, advocacy, and other competencies that contribute to positive youth development (Mawn et al., 2015; National Academies of Sciences, Engineering, and Medicine, 2019).

Despite these benefits, sustained and meaningful youth engagement remains a challenge. Youth engagement is often limited to advisory roles with minimal decision-making power (Kang et al., 2024). Few systems are in place to provide consistent support for their involvement. Opportunities for compensation, mentorship, and leadership development are limited and inconsistently implemented. These challenges tend to impact youth from historically marginalized communities the most, who may face heightened barriers such as adultism, transportation, or inflexible program schedules (McCabe et al., 2023; Kang et al., 2024). 

Recent studies describe practical strategies for meaningful youth engagement. Co-design approaches that involve youth from the beginning and throughout the project lifecycle are considered best practice (McCabe et al., 2023). Knowles et al. (2022) emphasize the value of building trust, offering flexible roles, and avoiding one-time consultation sessions. Programs that include mentorship, leadership training, and fair compensation are more likely to foster meaningful and lasting involvement. Critically, youth must be integrated into core decision-making processes, not just invited to provide input after plans have been developed (Kang et al., 2024).

### Current Study Aims

There are three specific aims of the current study and program report. The first is to describe the *Youth Leadership Academy* process and activities, which offers a replicable structure for embedding youth into CSMH teaming processes. The authors collected data from the youth participating in the YLA for formative evaluation purposes. The second aim is to report on evaluation findings concerning youth contributions to CSMH through summarizing youth-driven MTSS goals and action plans. Finally, we will report findings from pre-post analyses that shed light on changes in student-reported indicators of positive youth development during their participation in the YLA. 

## 2. YLA Program Delivery

### 2.1. YLA Background, Purpose, & Structure

The annual YLA is a national partnership program with middle and high school student leaders from across the U.S. who participate on their school CSMH teams, co-develop and implement school mental health initiatives in their school communities, and receive leadership training and support. The YLA was developed and facilitated by faculty and staff at the National Center for School Mental Health (NCSMH) and the Collaborative for Academic Social and Emotional Learning (CASEL). The YLA placed youth in central roles on CSMH teams, offering stipends, structured mentorship, and leadership development (described in detail below). Youth were actively engaged in setting goals and action plans to advance emotional well-being in schools. YLA participants are integrated within key components of CSMH system-building processes, including MTSS development, Learning Collaboratives (LCs), and continuous quality improvement efforts. The YLA models the highest levels of youth engagement described by Hart (1992), where young people and adults collaborate in shared decision-making.

The YLA was embedded within a national Learning Collaborative (LC) of CSMH teams, including professionals from state education agencies and K-12 districts and schools between the 2022 and 2025 academic years. The purpose of the LC was to support participating teams in implementing high-quality CSMH systems aligned with the Whole School, Whole Community, Whole Child (WSCC) model (Centers for Disease Control and Prevention, 2015). Each CSMH team participated in monthly virtual LC calls that involved training on topics related to school mental health, including best practices for meaningful youth engagement. They also received ongoing technical assistance from national experts to support each team with their CSMH quality improvement goals and action plans. Several participating LC teams recruited youth representatives to contribute to CSMH teaming processes via participation in the YLA.

The goals of the YLA were three-fold. They were (1) to enhance youth leadership capacities to serve as leaders in promoting emotional well-being in schools, (2) to integrate youth into CSMH teaming processes, where they provide input on goals and priorities in their state, district, and/or school-level CSMH teams and (3) to support youth with setting and leading the implementation of a student-driven CSMH-related goal.

The YLA involved eight hours of structured youth-centered virtual training and engagement facilitated by a team of national experts in school mental health and social and emotional learning. The eight hours included a four-hour YLA Summit and four one-hour YLA Action Calls. The Summit served as an orientation for youth in the fall. The objective of the Action Calls was to support students with setting and implementing an MTSS goal during the winter and spring months. All YLA sessions were held during school hours. Students primarily participated in school under the supervision of a school staff member who ensured they had access to a device and internet.

### 2.2. YLA Program Components

**Figure 1:**
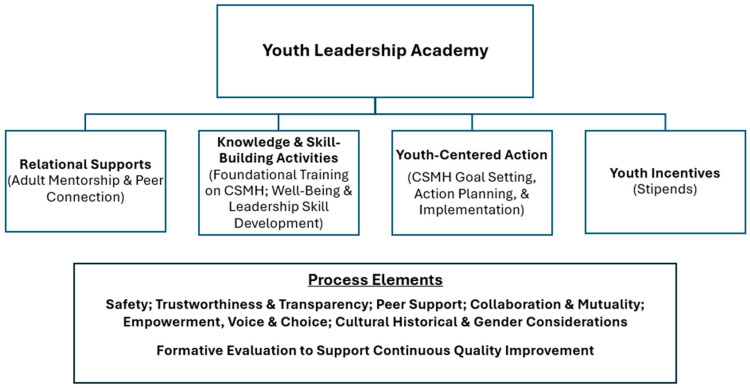
Youth Leadership Academy Components and Process Elements.

The YLA supported youth leader integration into CSMH systems’ building processes by offering students four programmatic components (relational supports, knowledge and skill-building activities, youth-centered action, and youth incentives). These components, along with important process elements, are described in the narrative below and depicted in Figure 1. 

#### 2.2.1. Relational Supports

During the YLA, youth received relational supports (e.g., mentorship, peer connection) from adults and peers. Participating districts and schools identified one staff member (e.g. school counselor, assistant superintendent, school principal) to be a District Youth Liaison (DYL) without any specific requirements about who could take on this role. DYLs often included school-employed professionals, like principals and school counselors, who had influence on decisions about implementation of youth ideas in their schools. DYLs were responsible for participating in all YLA activities and providing on-going mentorship to students to assist them in setting and implementing their MTSS goals both during and between structured YLA sessions. DYLs were typically a part of the CSMH team that participated in the LC that set and implemented CSMH team quality improvement goals. Relatedly, DYLs often served as youth engagement champions, where they worked to ensure student perspectives were integrated in CSMH priorities. All DYLs were invited to attend monthly LC trainings focused on various domains of school mental health, including youth engagement. While training was typically well attended by DYLs, attendance was not closely tracked. 

To foster peer connection, students were regularly engaged in community connector (icebreaker) activities, where youth shared about their personal interests, experiences, and strengths. The facilitators also engaged youth in structured activities to provide and receive support and constructive feedback to or from peers related to their goals. 

#### 2.2.2. Knowledge- and Skill-Building Activities

The program leaders prepared youth for active participation in CSMH teams by facilitating lessons and skill-building activities. Youth received developmentally appropriate didactics on topics related to CSMH and relevant frameworks. All didactics centered four priorities across CSMH-related frameworks (e.g., WSCC, MTSS, SEL), including supportive, responsive relationships; intentional learning experiences and interventions; skills development; and emotionally supportive environments that promote belonging. These priorities were abbreviated using the acronym “RISE” throughout the YLA. Additionally, participants regularly engaged in guided well-being activities (e.g., deep breathing, mindful movement, positive affirmations) to support focus and wellness for all at the start of each call.

To further enhance student knowledge and comfort with foundational topics regarding CSMH, they were engaged in interactive breakout group discussions. Most discussions occurred in small breakout rooms of 3 to 8 youth to allow each student many opportunities to speak. During discussions, the facilitators supported adherence to community norms and ensured that each student had an opportunity to speak if desired. Students responded to discussion prompts first through taking informal notes on their responses prior to sharing their ideas aloud. This process aimed to allow youth to thoughtfully generate their responses before sharing their reflections with the group.

Youth also regularly participated in leadership skill-building activities to enhance their confidence and capacity to serve as leaders. These activities included giving a professional introduction, delivering an “elevator speech” on their interest in school mental health, using assertive communication skills, giving and receiving constructive feedback, goal setting, and seeking mentorship. To optimize skill acquisition and enhance youth comfort with using skills, youth engaged in skill-based role-plays and practice sessions prior to being invited to use new skills during the academy.

#### 2.2.3. Youth-Centered Action 

Throughout the YLA, DYL, and other adult CSMH team members joined students from their state in small breakout rooms where students were engaged in conversations with their state, district, and/or school leadership to inform MTSS goal setting and action planning. During Action Calls, youth set and received support with planning a youth-driven MTSS goal of their choice. Youth leaders used an adapted “SMART” (specific, measurable, achievable, realistic, time-bound) framework (Doran, 1981) to organize their goals. DYLs and all CSMH team members were invited to participate in youth-centered CSMH teaming processes throughout the YLA to seek student input on school mental health priorities, goals, and action plans.

#### 2.2.4. Youth Incentives

Youth participation was incentivized with stipends ($20 gift cards for each hour of engagement in structured YLA activities). Youth participation was voluntary, and they received stipends even if they elected not to participate in various components of the program (i.e., goal setting, discussions, etc.).

#### 2.2.5. The YLA Process of Engagement

The purpose of the current project report was to describe the structure, process and outcomes of a Youth Leadership Academy embedded within CSMH systems teams. YLA facilitators used an intentional process to inspire youth as co-decision-makers and collaborators with adults. The facilitators used best practices for meaningful youth engagement that are aligned with the Substance Abuse and Mental Health Services Administration (SAMHSA, 2014) trauma-informed principles as follows: fostering emotional and physical safety, establishing and maintaining trust and transparency in relationships, creating opportunities for peer support and connection, adults working collaboratively with youth, focusing on adults sharing power with youth (i.e., via use of shared decision-making processes), and fostering emotionally safe environments that promote belonging for all students.

Community norms were co-created with youth to foster a safe and inclusive space conducive to connection, learning, and youth empowerment, voice, and choice during the YLA. The group reviewed these norms at the beginning of each call. Program evaluation efforts were used to support formative evaluation of the YLA. Throughout all years, both youth and adult participants provided feedback on the YLA through brief anonymous surveys following each session. The findings from these evaluation surveys were regularly reviewed to enhance the quality of the program.

## 3. Materials and Methods

### 3.1. Participants 

Youth leaders (*N* = 114) between 6th and 12th grades participated in the year-long *Youth Leadership Academy* implemented annually between 2022 and 2025. A total of 42 students participated in year 1, the same number (42) were engaged in year 2, and there were 30 participants in year 3. While the YLA was intended to be a one-year program, four youth chose to participate in two consecutive years of the YLA to continue working on their goals. During years 1 and 2, minimal youth demographic data, outside of student grade levels, was collected, as it was not essential information within the scope of the initial evaluation plan. In year three, the program evaluation efforts were expanded and included more robust demographic items (e.g., race/ethnicity, gender identity, age). Twenty-five of the 30 youth leaders in year 3 completed the core SEL measure that included demographic questions. Most (72%) respondents were high-school-aged (*M* = 15.32, *SD* = 1.87) and girls (64%). Youth-reported racial/ethnic identities included “American Indian, Indigenous, Native American or Alaskan Native” (40%), “Hispanic or Latin(o)(x)” (20%), “White” (12%), “Asian (including South Asian/Indian)” (4%), “Black or African American” (4%), and 20% of students selected two or more of the racial/ethnic categories. 

Youth participants were enrolled in schools within one of the thirteen states involved in the national LC, in which the YLA was embedded. These states represented several major regions of the U.S., including Northeastern, Midwestern, Southern, and Western regions. There were 38 school districts represented in the LC. Districts were diverse in terms of urbanicity and size. About 47% are rural, 26% suburban, and 24% urban, and 39% are small in size (<3000 students), 29% medium (between 3000 and 10,000 students), and 32% large (>10,000 students). A total of 33 districts (across 12 states) had youth participating in the YLA during years 1-3. These districts were diverse in urbanicity and size. About 52% are rural, 18% urban, and 30% suburban, and 36% are small in size, 30% medium, and 33% large. 

### 3.2. Recruitment

The District Youth Liaisons recruited 1 to 3 youth leaders (per district) by soliciting nominations by school staff (e.g., teachers, counselors). Given the focus of the overarching LC to help schools address chronic disease by promoting emotional well-being in schools, the DYLs selected a diverse group of youth, including students from communities most impacted by chronic disease and its risk factors (i.e., youth of color and those living in poverty). It was recommended that youth selected also exhibited leadership potential, as determined by a known or expressed passion for or interest in school mental health and openness to contributing their perspectives on emotional well-being in schools. These recommendations for youth selection were made to ensure they would have the capacity and interest to fully participate in the YLA activities.

### 3.3. Procedures

During each year of the annual, 8-hour YLA (described in the Introduction), an evaluation process was implemented. It involved both youth and adult participants providing on-going feedback via voluntary online, anonymous surveys. In years 1 through 3, both youth and adults completed evaluation surveys after each YLA session, including the two-part YLA Summit facilitated in the fall semester and the four YLA Action Calls, facilitated in the winter/spring semester. In year 3, a pre-post evaluation design was employed, where students completed a questionnaire concerning their core SEL skills. The pre-survey administration occurred at the beginning of the first YLA session (prior to initiation of core program activities) and the post-questionnaire was completed at the end of the final session. To allow the evaluators to match data for pre-post responses, and maintain respondent anonymity, youth used alpha-numeric codes on their surveys at both time points. Only a selection of YLA evaluation data was used for this project report, aligned with the aims of the manuscript. 

### 3.4. Ethical Considerations

The university IRB determined this program evaluation did not constitute human subjects research. As such, a standard research informed consent process was not required as a part of the evaluation. However, youth and their parents/legal guardians provided written permission and youth agreement forms to participate in the YLA activities and program evaluation process prior to the start of activities. Youth participation was voluntary. Youth participation was incentivized through electronic gift cards at a rate of $20 per hour of engagement in structured YLA sessions (8 hours total). To protect youth confidentiality, no identifying data was collected via surveys and correspondingly all data files do not include identifying data. 

### 3.5. Measures

#### 3.5.1. Youth SMART Goal Questionnaire

Youth participants completed a standardized SMART goal-setting questionnaire designed to guide them in developing a student-directed goal to promote emotional well-being in schools aligned with an adapted SMART framework (Doran, 1981). Students established a goal of their intention to lead/co-lead or support the planning, implementation, or evaluation of an MTSS service or support. The SMART goal form prompted students to write the goal and respond to seven structured items to reflect with qualitative responses how their goal is specific, measurable, achievable, relevant, time-bound, and fosters emotionally safe environments that promote belonging for all. Youth or DYLs submitted student SMART goals between the first and third YLA Action Calls. 

#### 3.5.2. Core Social and Emotional Skills Survey

Youth leaders completed a pre-post emotional and social skills survey (Hello Insight, 2021) via an online platform during year 3 of the YLA. This survey uses 22 items to capture the domains of Academic Self-Efficacy (4 items), Contribution (to community) (4 items), Positive Identity (4 items), Self-Management (6 items), and Social Skills (4 items), among other items. Scores are generated for core emotional and social skills overall, in addition to the listed domains. Youth rated statements on a 5-point Likert-type scale with response options varying between agreement, fit of statement to self, count, and frequency. Sample items include “I know how I can use my interests and skills to make my community better,” (Contribution domain), “I take the time to find out about my own identities,” (Positive Identity domain), “I am confident I can do well in school,” (Academic Self-Efficacy domain), “I can stay calm even when things get stressful,” (Self-Management domain) and “Other people’s feelings matter to me,” (Social Skills domain).” Psychometric analyses on a sample of over 15,000 young people supported construct validity via confirmatory factor analysis and demonstrated reliability with strong internal consistency with Cronbach’s alphas for each domain ranging from α = 0.73 to α = 0.80 (Hello Insight, 2021).

## 4. Results

### 4.1. Youth Leader Multi-Tiered System of Support Contributions

All youth leaders were invited to submit a goal of their choice related to promoting emotional well-being in schools. Goals reflected student intentions to lead/co-lead or support the implementation of a school mental health service or support across any of the three MTSS tiers. Youth were invited to lead or co-lead a goal with other youth leaders. Eighty-two students (72% of all participants) submitted goals. Fifty-eight unduplicated goals were included in analyses conducted to address the study’s first aim of describing youth leader contributions to MTSS.

#### 4.1.1. Thematic Analysis of Youth-Driven MTSS Goals

A thematic analysis approach was used to organize student goals within predetermined themes (i.e., MTSS tiers: Tier 1-Universal Mental Health Promotion/Prevention, Tier 2-Early Intervention, or Tier 3-Treatment). This method allowed systematic identification, analysis, and interpretation of themes across qualitative goal data. First, all student-identified goals were reviewed in their entirety to ensure familiarity with the content. Goals were then grouped into broad themes based on the aim of the goal to lead/co-lead or support universal mental health promotion/prevention supports at the classroom-, grade-, and/or whole-school levels (coded Tier 1); early intervention supports for students with increased risk for mental health challenges or already existing mild difficulties (Tier 2); or treatment for students with existing mental health conditions (Tier 3). 

After coders sorted youth goals into tiers, they were reviewed in their entirety and coded deductively, being assigned one or more subtheme code predetermined by common domains within MTSS tiers. These domains were informed by the NCSMH quality guides on MTSS (NCSMH, 2023a, 2023b). In a few cases, single instances of domains were coded as subthemes because they aligned with a common MTSS target (e.g., educator well-being within Tier 1, and group counseling and peer mentoring within Tier 2). Two coders, who are researchers with deep knowledge in MTSS and experienced in qualitative methods in education and psychology, reviewed all goals to ensure coding reliability and resolved discrepancies through discussion and consensus. This method ensured both rigor and alignment with the MTSS framework to support a comprehensive summary of youth contributions to MTSS.

Nearly all (97%) of the 58 student-driven goals focused on supporting Universal Mental Health Promotion and Prevention activities (Tier 1). Two student goals focused on implementing Early Intervention (Tier 2) in schools. None of the student goals related to school-based treatment (Tier 3) services. There were five subthemes that emerged across Tier 1 goals. These subthemes reflected common targets of Tier 1 supports, specifically mental health literacy (MHL), school climate, educator professional development (PD), social and emotional learning (SEL), and educator well-being. Student goals reflected two domains (subthemes) of Tier 2 supports, including small group counseling and peer mentoring. Table 1 reflects the frequencies of subthemes within each tier. The following narrative summarizes student MTSS goals.

#### 4.1.2. Mental Health Literacy 

MHL enhances understanding of mental health problems and strategies to promote well-being, help-seeking, and stigma reduction (Kutcher et al., 2016). Overall, youth leader MHL goals focused on developing resources that increase awareness of and/or strategies to cope with mental health difficulties, specifically substance use (e.g., drinking alcohol, vaping), anxiety, and stress. Youth also set out to enhance student awareness of ways to seek help and healing concerning exposure to trauma and adversity, specifically bullying and sexual abuse. Some goals included an intentional aim to give students opportunities to practice various well-being skills such as yoga, mindfulness, deep breathing, and artistic expression.

Youth identified various methods of disseminating MHL information and resources. These methods included sharing via websites, school magazines, flyers, pamphlets, infographics, posters, and social media posts. An innovative strategy to promote mental health literacy involved use of *Claymation*, where figures are created out of clay and several photographs are taken to display a story or narrative—in this case, about important MHL messaging. Some youth established plans to enhance their peers’ understanding of mental health difficulties and exposure to various well-being practices at structured events and/or activities for the school community, including wellness clubs, recurring well-being breaks for students (e.g., during study hall), and district-wide health fairs.

#### 4.1.3. School Climate 

Youth leaders developed goals and action plans to promote a positive school climate. Many students planned to continue supporting or enhancing their involvement in Tier 1 supports aimed at improving relationships focusing on student-student and student-teacher relationships. They established plans to implement activities during the school day that would allow young people and staff to connect through shared interests and experiences. Some students implemented “kindness awareness” campaigns to promote acts of kindness by rewarding prosocial acts with public recognition. Many youth set goals to make their school’s physical environment more uplifting and supportive by posting inspirational quotes and positive affirmations around the building as well as messaging to enhance inclusivity and belonging.

Students aimed to promote a positive school climate by enhancing youth and family engagement. These goals focused on enhancing opportunities for young people and families to learn about mental health. Additionally, students established plans to obtain youth and family input on school policies, practices, and programming through administering youth- and family-centered surveys and student leadership opportunities. 

#### 4.1.4. Educator Professional Development

Two youth goals aimed to promote well-being by enhancing educator skills through student-informed PD. One student devised a plan to collaborate with district leaders to establish educator PD to train staff on culturally responsive practices to prevent identity-based harm and discrimination. Another student worked toward training teachers on the impact of their practices on student well-being. They also planned to integrate content on how educators can elevate student voice in classrooms. 

#### 4.1.5. Social and Emotional Learning

Social and emotional learning was the focus for several goals. During the YLA, students implemented an “afterschool counseling group” for all students who were interested in developing various social and emotional skills such as displaying empathy and promoting health equity. Another student established a plan to identify local businesses to provide SEL and life skills (i.e., entrepreneurship skills) training to students to prepare young people for the workforce. 

#### 4.1.6. Educator Well-Being

One youth goal focused on providing supports for educators’ well-being. The student goal was to establish a self-care bulletin board and coordinate weekly self-care activities to promote positive mental health for students and staff.

#### 4.1.7. Small Group Counseling

Small group counseling is a common service within Tier 2. Small group counseling is offered to students based on increased risk for mental health concerns, including interventions for youth experiencing distress related to identity-based discrimination or being in non-affirming environments. One youth leader goal focused on offering a Tier 2 group for their LGBTQ peers who have been bullied or excluded to give them opportunities to discuss issues impacting their well-being and receive affirming supports. 

#### 4.1.8. Peer Mentoring

One youth leader established a goal to personally serve as a mentor to their peers when they experience mental health related challenges or concerns at school. In their planned role, the student would connect their peers to available mental health supports at school. 

### 4.2. Youth Leader Positive Youth Development

To assess the impact of participation in the YLA on indicators of positive youth development, youth leaders completed the pre-assessment (*n* = 32) and post-assessment (*n* = 25) from the Core Social and Emotional Learning (SEL) survey during year 3 of the YLA. Pre-test and post-test averages from all respondents are displayed in Table 2.

Only 25 youth completed paired pre-post assessments, and three domains (Academic Self-Efficacy, Positive Identity, Social Skills) did not have normally distributed scores. Therefore, evaluators used the non-parametric Wilcoxon signed-rank tests in conjunction with Cliff’s δ (Meissel & Yao, 2024) to identify and interpret changes. Youth leaders reported significant increases in their Core SEL skills with a small effect. At the domain level, youth leaders also reported increases in Contribution and Positive identity, both with small effects. Table 3 provides the values for all tests.

## 5. Discussion

This project report describes the structure and process of the YLA that offers a replicable structure for embedding youth leaders into CSMH system-building processes. Youth participants had opportunities to co-develop and implement school mental health initiatives by being elevated to central roles on CSMH teams and being provided stipends, structured mentorship, and leadership development. The YLA models the highest levels of youth engagement described by Hart (1992), where young people and adults collaborate in shared decision-making on setting priorities and youth goals reflect youth-driven and initiated goals and action plans. 

The YLA program address critiques of early initiatives where youth engagement was either non-existent or limited such that young people were siloed, manipulated, and/or tokenized. Lack of youth perspective in processes to build and improve school-based services denies students an opportunity to inform and evaluate the very supports designed to serve them. Alternatively, meaningful youth engagement can help schools establish services that are relevant and responsive to youth’s stated needs and preferences (Dolaty et al., 2025). The description of the YLA program delivery can be used by schools and districts to leverage the specialized expertise of young people to build comprehensive school-based systems of support for mental health that center student perspectives and preferences.

### 5.1. Youth Contributions to MTSS 

A primary goal of the current study was to explore YLA participant contributions to CSMH system-building, specifically MTSS goals and action plans. Findings revealed that students primarily contributed to Tier 1 (Universal Mental Health Promotion and Prevention supports), selecting goals that covered a breadth of common Tier 1 domains including mental health literacy, social and emotional learning, school climate, and educator well-being and PD. Tier 2 (Early Intervention services) were prioritized among a smaller subset of students. 

This breadth and depth of student MTSS contributions, specifically regarding Tier 1, is significant given the evidence of the positive impacts of Tier 1 strategies on desirable student outcomes. For instance, universal mental health supports are related to increases in student help-seeking and coping, increased mental health knowledge, and improved social and emotional well-being (O’Connor et al., 2018). Positive school climate activities enhance student perceptions of belonging and are related to improvements in student academic achievement (Cheryan et al., 2014; Guardino & Fullerton, 2010). Relatedly, school-based supports that otherwise reinforce prosocial behaviors help to reduce bullying behaviors (McCarty et al., 2016; González Moreno & Molero Jurado, 2024). Additionally, SEL skill development activities have been shown to promote academic and SEL competencies for children and adolescents (Durlak et al., 2011). As such, high-quality implementation of YLA youth leader-driven goals is likely to positively impact school safety, emotional well-being and academic success for participating schools. 

While youth leaders in the current study made smaller contributions to Tier 2 (Early Intervention) supports, compared to Tier 1, their efforts in this domain are important as they reflect services designed to support students with elevated risk for mental health conditions to prevent the onset or worsening of student distress. Additionally, the YLA Tier 2-related goals centered the provision of affirming care for LGBTQ+ youth who often experience harassment, exclusion, and violence that increase mental health risk through adulthood (Copeland et al., 2013). Other Tier 2 goals focused on the provision of peer mentoring, which have positive psychological effects on both youth facilitators and students receiving support (Douglas et al., 2019; Felter et al., 2023). Therefore, the identified Tier 2 goals and action plans, if implemented well, are likely to have positive impact on the well-being of youth impacted by marginalization and adversity. 

Educator well-being and professional development were also a smaller focus for emotional well-being promotion in schools among the YLA participants. However, these focuses are critical to the success of school-based supports, as educators that are emotionally well and well-trained help to foster school environments that promote student well-being and success (Schonert-Reichl, 2017). 

Notably none of the youth goals focused on positive disciplinary practices. It may not have been immediately evident to young people how school discipline relates to student mental health. Youth leaders may benefit from future opportunities to learn about this connection and inform school disciplinary practices.

### 5.2. Youth Positive Development 

An additional primary goal of the current study was to explore the impact of the YLA on indicators of positive youth development. Findings from pre-post YLA evaluation surveys indicated that youth leaders experienced growth in core SEL competencies during year 3 of the academy. Youth leaders reported significant positive changes in contribution, which refers to a young person’s desire to engage with and contribute to family, community, and society. The benefit of this outcome is that contribution is related to civic engagement during adulthood and improved social development (Lerner et al., 2005). Youth leaders also reported significant growth in positive identity, an indicator of positive youth development that is associated with higher confidence and reduced behavioral problems (McLaughlin, 2000). 

There are several elements of the YLA that may have contributed to growth in youth skills. Youth leaders participated in CSMH teaming processes where they contributed their perspectives to CSMH systems planning, implementation, and evaluation, which potentially contributed to growth contribution and positive identity. This interpretation is supported by experimental findings that active youth leadership and civic engagement are associated with significant growth in positive youth development skills such as critical thinking, self-efficacy, and civic engagement (Ozer & Douglas, 2013). Additionally, during the academy, adult facilitators and DYLs frequently offered individualized and global praise to youth for their input and progress toward their goals. Students also engaged in activities where they shared their personal, familial, and community strengths. It is plausible that these activities contributed to students’ own positive perceptions of themselves, as adult social support in families, schools, and communities is associated with positive youth identity (Jankowska-Tvedten & Wiium, 2023).

There were no significant changes in student reported self-management, academic self-efficacy, or social skills. Lack of growth in academic self-efficacy is logical given academic motivation and performance were not of focus in the YLA. Regarding social skills, the corresponding items on the pre-post survey focused on youths’ abilities to integrate others’ perspectives and to develop care and empathy. While the YLA focus was to engage students in informing school-based services, which required them to consider the perspectives and needs of their peers, there were limited activities targeting student empathy building. However, students were actively engaged in other social skills training such as assertive communication and giving and receiving constructive feedback, and no items on the pre-post survey assessed these skills. 

There are notable limitations to the current study design that warrant caution when interpreting relationships between program participation and skill development. First, only twenty-five of 114 youth leaders completed both pre- and post- core SEL assessments, which limits the representativeness of these findings. Relatedly, the original purpose of the evaluation procedure, including tracking of student skills over time was to support formative evaluation. Relatedly, students were selected based on their interest and potential to support emotional well-being in schools; they were not systematically recruited to represent a broader population. This thus limits the generalizability of study findings. Additionally, growth in student-reported contribution and positive identity may reflect natural changes in these skills over time. Given the students’ pre-existing leadership potential and experiences, they may have opportunities throughout the school year, unrelated to the YLA, to develop these skills. This study also lacked a control group against which to compare changes in these skills over time, which would increase internal validity.

Further research is warranted to enhance understanding of the impact of youth leadership on advancing emotional well-being in schools as well as how the YLA impacts positive youth development. Future programs should track the implementation of students’ goals to better understand their impact on school mental health outcomes. Relatedly, experimental program component analysis studies would be useful in understanding how various YLA components contribute to positive youth development. Future studies may also seek to adapt the YLA curriculum to engage youth with disabilities, elementary-aged students, and English language learners. Ideally, replications of the YLA involve young people serving as co-facilitators of programmatic activities (e.g., skills training, small group discussions) to explore how young facilitators and youth participants may benefit from a peer-led YLA.

## 6. Conclusions

The YLA offered youth leaders preparation (i.e., knowledge and skills-building activities), supports (i.e., adult mentorship and peer connection, stipends), and opportunities to inform emotional well-being supports in schools. Overall, the study findings suggest that meaningful youth engagement in CSMH systems development efforts can foster meaningful partnerships between adults and young people. Youth offer adult leaders insightful goals and strategies to promote emotional well-being in schools, while young people experience opportunities to lead and develop their knowledge and skills. Additionally, initiatives that empower youth leadership in CSMH may function as an early pipeline to build the school mental health workforce. 

The YLA offers a structure for embedding youth in multi-level school mental health teaming processes to build CSMH systems. The YLA can inform state- and district-level policies to institutionalize meaningful youth engagement and prevent tokenized or siloed participation. To support this priority, states and districts are challenged to explore sustainable funding and partnerships to support the continued growth and expansion of programming to elevate youth perspectives and leadership in CSMH system-building processes.

## Figures and Tables

**Table 1:** Instances of Subthemes (Focus Areas) for Youth-Driven MTSS Goals.

Subtheme (MTSS Domain)	*Frequency*
*Tier 1: Universal Mental Health Promotion/Prevention*	
Mental Health Literacy	30
School Climate	25
Educator PD	2
Social and Emotional Learning	2
Educator Well-Being	1
*Tier 2: Early Intervention*	
Small Group Counseling	1
Peer Mentoring *	1
*Tier 3: Treatment*	
Not applicable	-

* Peer mentoring in this example was specifically intended for youth at increased risk for mental health concerns.

**Table 2:** Average Pre-Post Scores from Core SEL Survey (All Respondents).

Domain	Pre-Test *M*	Post-Test *M*
Core Emotional and Social Skills	83.88	86.81
Academic Self-Efficacy	90.39	93.37
Contribution	78.08	81.36
Positive Identity	81.46	86.72
Self-Management	80.22	83.42
Social Skills	90.78	90.24

**Table 3:** Wilcoxon Signed-Rank Test and Cliff’s Delta Results from Pre-Post Core Emotional and Social Skills Survey (Matched Responses Only).

Domain	Pre-Test *M*	Post-Test *M*	*z*	*p*	δ
Core Emotional and Social Skills *	83.38	86.81	2.34	0.02	0.22
Academic Self-Efficacy	90.52	93.37	1.50	0.13	0.22
Contribution *	77.51	81.36	2.06	0.04	0.20
Positive Identity *	80.26	86.72	1.98	0.04	0.28
Self-Management	79.34	83.42	1.49	0.14	0.28
Social Skills	90.96	90.24	−0.11	0.91	−0.09

* Significant difference (*p* < 0.05) with small effect.

## Data Availability

The data presented in this study are available on request from the corresponding author. The data are not publicly available due to confidentiality agreements with participants.

## Abbreviations

The following abbreviations are used in this manuscript:

CSMH	Comprehensive School Mental Health
LC	Learning Collaborative
DYL	District Youth Liaison
MHL	Mental Health Literacy
MTSS	Multi-Tiered System of Support
NCSMH	National Center for School Mental Health
PD	Professional Development
SEL	Social and Emotional Learning
RISE	Supportive, responsive Relationships; Intentional learning experiences and interventions; Skills development; and Emotionally supportive environments that promote belonging
SMART	Specific, Measurable, Achievable, Realistic, Time-Bound
WSCC	Whole School, Whole Community, Whole Child
YLA	Youth Leadership Academy

## References

[B1-behavsci-15-01563] Bohnenkamp J., Robinson P., Connors E., Carter T., Orenstein S., Reaves S., Hoover S., Lever N. (2024). Improving school mental health via national learning collaboratives with state and local teams: Components, feasibility, and initial impacts. Evaluation & the Health Professions.

[B2-behavsci-15-01563] Centers for Disease Control and Prevention (2015). About the Whole School, Whole Community, Whole Child (WSCC) model.

[B3-behavsci-15-01563] Cheryan S., Ziegler S. A., Plaut V. C., Meltzoff A. N. (2014). Designing classrooms to maximize student achievement. Policy Insights from the Behavioral and Brain Sciences.

[B4-behavsci-15-01563] Colomeischi A. A., Duca D. S., Bujor L., Rusu P. P. (2022). Impact of a school mental health program on children’s and adolescents’ socio-emotional skills and psychosocial difficulties. Children.

[B5-behavsci-15-01563] Copeland W. E., Wolke D., Angold A., Costello E. J. (2013). Adult psychiatric outcomes of bullying and being bullied by peers in childhood and adolescence. JAMA Psychiatry.

[B6-behavsci-15-01563] Dolaty S., Midouhas E., Deighton J., Somerville M. P. (2025). Public participation in mental health programming: Insights into the ways young people are involved in the development, delivery, and evaluation of mental health initiatives in school and community spaces. International Journal of Adolescence and Youth.

[B7-behavsci-15-01563] Doran G. T. (1981). There’s a SMART way to write management’s goals and objectives. Management Review.

[B8-behavsci-15-01563] Douglas L. J., Jackson D., Woods C., Usher K. (2019). Rewriting stories of trauma through peer-to-peer mentoring for and by at-risk young people. International Journal of Mental Health Nursing.

[B9-behavsci-15-01563] Durlak J. A., Weissberg R. P., Dymnicki A. B., Taylor R. D., Schellinger K. B. (2011). The impact of enhancing students’ social and emotional learning: A meta-analysis of school-based universal interventions. Child Development.

[B10-behavsci-15-01563] Fein E. H., Agbangnin G., Murillo-León J., Parsons M., Sakai-Bismark R., Martinez A., Gomez P. F., Chung B., Chung P., Dudovitz R., Inkelas M., Kataoka S. (2023). Encouraging “positive views” of mental illness in high schools: An evaluation of Bring Change 2 Mind youth engagement clubs. Health Promotion Practice.

[B11-behavsci-15-01563] Felter J., Chung H. L., Guth A., DiDonato S. (2023). Implementation and outcomes of the Trauma Ambassadors Program: A case study of trauma-informed youth leadership development. Child and Adolescent Social Work Journal.

[B12-behavsci-15-01563] Golberstein E., Wen H., Miller B. F. (2023). Effects of school-based mental health services on youth outcomes. Journal of Human Resources.

[B13-behavsci-15-01563] González Moreno A., Molero Jurado M. d. M. (2024). Intervention programs for the prevention of bullying and the promotion of prosocial behaviors in adolescence: A systematic review. Social Sciences & Humanities Open.

[B14-behavsci-15-01563] Guardino C. A., Fullerton E. (2010). Changing behaviors by changing the classroom environment. Teaching Exceptional Children.

[B15-behavsci-15-01563] Hart R. A. (1992). Children’s participation: From tokenism to citizenship.

[B16-behavsci-15-01563] Hello Insight (2021). HI SEL: Technical report summary.

[B17-behavsci-15-01563] Hoover S., Bostic J. (2021). Schools as a vital component of the child and adolescent mental health system. Psychiatric Services.

[B18-behavsci-15-01563] Hoover S., Lever N., Sachdev N., Bravo N., Schlitt J., Acosta Price O., Sheriff L., Cashman J. (2019). Advancing comprehensive school mental health: Guidance from the field.

[B19-behavsci-15-01563] Jankowska-Tvedten A., Wiium N. (2023). Positive youth identity: The role of adult social support. Youth.

[B20-behavsci-15-01563] Kang E., Kindler C., Saint Amour A. T., Locus K., Hosaka K. R. J., Leslie M. C., Patel N. A. (2024). Youth engagement synergy in mental health legislation and programming. Child and Adolescent Psychiatric Clinics of North America.

[B21-behavsci-15-01563] Knowles S., Sharma V., Fortune S., Wadman R., Churchill R., Hetrick S. (2022). Adapting a codesign process with young people to prioritize outcomes for a systematic review of interventions to prevent self-harm and suicide. Health Expectations.

[B22-behavsci-15-01563] Kutcher S., Wei Y., Coniglio C. (2016). Mental health literacy: Past, present, and future. Canadian Journal of Psychiatry. Revue Canadienne de Psychiatrie.

[B23-behavsci-15-01563] Lerner R. M., Lerner J. V., Almerigi J. B., Theokas C., Phelps E., Gestsdottir S., Naudeau S., Jelicic H., Alberts A., Ma L., Smith L. M., Bobek D. L., Richman-Raphael D., Simpson I., Christiansen E. D., von Eye A. (2005). Positive youth development, participation in community youth development programs, and community contributions of fifth-grade adolescents: Findings from the first wave of the 4-H study of positive youth development. The Journal of Early Adolescence.

[B24-behavsci-15-01563] Lever N., Orenstein S., Jaspers L., Bohnenkamp J., Chung J., Hager E. (2024). Using the whole school, whole community, whole child model to support mental health in schools. Journal of School Health.

[B25-behavsci-15-01563] Mawn L., Welsh P., Stain H. J., Windebank P. (2015). Youth Speak: Increasing engagement of young people in mental health research. Journal of Mental Health.

[B26-behavsci-15-01563] McCabe E., Amarbayan M. M., Rabi S., Mendoza J., Naqvi S. F., Thapa Bajgain K., Zwicker J. D., Santana M. (2023). Youth engagement in mental health research: A systematic review. Health Expectations.

[B27-behavsci-15-01563] McCarty S., Teie S., McCutchen J., Geller E. S. (2016). Actively caring to prevent bullying in an elementary school: Prompting and rewarding prosocial behavior. Journal of Prevention & Intervention in the Community.

[B35-behavsci-15-01563] McLaughlin M. W. (2000). Community counts: How youth organizations matter for youth development.

[B28-behavsci-15-01563] Meissel K., Yao E. S. (2024). Using Cliff’s delta as a non-parametric effect size measure: An accessible web app and R tutorial. Practical Assessment, Research & Evaluation.

[B29-behavsci-15-01563] Miles J., Espiritu R. C., Horen N., Sebian J., Waetzig E. (2010). A public health approach to children’s mental health: A conceptual framework.

[B30-behavsci-15-01563] National Academies of Sciences, Engineering, and Medicine (2019). The promise of adolescence: Realizing opportunity for all youth.

[B31-behavsci-15-01563] National Center for School Mental Health (NCSMH) (2023a). School mental health quality guide: Mental health promotion services and supports (tier 1).

[B32-behavsci-15-01563] National Center for School Mental Health (NCSMH) (2023b). School mental health quality guide: Early intervention and treatment services and supports (tiers 2 and 3).

[B33-behavsci-15-01563] O’Connor C. A., Dyson J., Cowdell F., Watson R. (2018). Do universal school-based mental health promotion programmes improve the mental health and emotional wellbeing of young people? A literature review. Journal of Clinical Nursing.

[B34-behavsci-15-01563] Ozer E. J., Douglas L. (2013). The impact of participatory research on urban teens: An experimental evaluation. American Journal of Community Psychology.

[B36-behavsci-15-01563] Schonert-Reichl K. A. (2017). Social and emotional learning and teachers. The Future of Children.

[B37-behavsci-15-01563] Substance Abuse and Mental Health Services Administration (2014). SAMHSA’s concept of trauma and guidance for a trauma-informed approach. *HHS Publication No. (SMA) 14-4884*.

[B38-behavsci-15-01563] Weist M. D., Eber L., Horner R., Splett J., Putnam R., Barrett S., Perales K., Fairchild A. J., Hoover S. (2018). Improving multi-tiered systems of support for students with “internalizing” emotional/behavioral problems. Journal of Positive Behavior Interventions.

